# Evaluación crítica y meta-análisis de estudios de variación biológica para albúmina glicosilada, glucosa y HbA_1c_

**DOI:** 10.1515/almed-2020-0040

**Published:** 2020-08-27

**Authors:** Carmen Ricós, Pilar Fernández-Calle, Elisabet Gonzalez-Lao, Margarida Simón, Jorge Díaz-Garzón, Beatriz Boned, Fernando Marqués-García, Joana Minchinela, Maria Carmen Perich, Xavier Tejedor-Ganduxé, Zoraida Corte, Aasne K. Aarsand, Berna Aslan, Anna Carobene, Abdurrahman Coskun, Sverre Sandberg

**Affiliations:** Spanish Society of Laboratory Medicine (SEQC_ML_), Analytical Quality Commission, Padilla, 323, Barcelona, España; SEQC_ML_, Analytical Quality Commission, Barcelona, España; EFLM, Task Group on Biological Variation Database, Madrid, España; EFLM, Working Group on Biological Variation, Madrid, España; Hospital Universitario La Paz, Madrid, España; Quality Healthcare, Grupo ACMS, Madrid, España; EFLM, Task Group on Biological Variation Database, Vilafranca del Penedes, España; Consortium of Laboratory Intercomarcal Alt Penedès and Garraf l’Anoia, Vilafranca del Penedes, España; Hospital Royo Villanova, Zaragoza, España; EFLM, Task Group on Biological Variation Database, Salamanca, España; Hospital Universitario de Salamanca, Salamanca, España; EFLM, TaskGroup on Biological Variation Database, Badalona, España; Hospital Germans Trias i Pujol, Badalona, España; EFLM, Task Group on Biological Variation Database, Barcelona, España; Hospital Vall d’Hebron, Barcelona, España; Hospital Universitario San Agustin, Aviles, España; EFLM, Task Group on Biological Variation Database, Bergen, Norway; EFLM, Working Group on Biological Variation, Bergen, Norway; Haukeland University Hospital, Bergen, Norway; Norwegian Quality Improvement of Laboratory Examinations, Haraldplass Deaconess Hospital, Bergen, Norway; EFLM, Task Group on Biological Variation Database, Toronto, Canada; Institute for Quality Management in Healthcare of Canada, Toronto, Canada; EFLM, Task Group on Biological Variation Database, Milan, Italy; EFLM, Working Group on Biological Variation, Milan, Italy; Laboratory Medicine, Ospedale San Raffaele, Milan, Italy; EFLM, Task Group on Biological Variation Database, Istanbul, Turkey; EFLM, Working Group on Biological Variation, Istanbul, Turkey; Acibadem Universitesi, Istanbul, Turkey; EFLM, Task Group on Biological Variation Database, Bergen, Norway; EFLM, Working Group on Biological Variation, Bergen, Norway; Department of Global Public Health, Bergen, Norway

**Keywords:** variación biológica, diabetes mellitus, base de datos de variación biológica

## Abstract

**Objetivos:**

A lo largo de los años se han publicado numerosos artículos sobre variación biológica (VB) de diferente calidad. Los objetivos de este trabajo fueron realizar una revisión sistemática y una evaluación crítica de los estudios de VB para albúmina glicosilada y proporcionar datos actualizados de VB para glucosa y HbA_1c_, incluyendo prestigiosos estudios recientemente publicados como el Estudio de Variación Biológica Europea (EuBIVAS).

**Métodos:**

Se hizo una búsqueda bibliográfica sistemática para identificar estudios sobre VB, encontrándose 9 estudios no incluidos en la primera revisión: 4 para albúmina glicosilada, 3 para glucosa y 3 para HbA_1c_. Se realizó una evaluación crítica de los estudios relevantes, utilizando la herramienta *Biological Variation Data Critical Appraisal Checklist* (BIVAC). Se obtuvieron los estimados globales de VB mediante meta-análisis de los estudios que cumplían los requisitos BIVAC, realizados en individuos sanos con estudios de diseño similar.

**Resultados:**

Un estudio recibió el grado A, dos el B y 6 el C. en la mayoría de los casos el grado C se asoció a deficiencias en el análisis estadístico de los datos. Los estimados de VB para albúmina glicosilada fueron: CV_I_ = 1,4%(1,2–2,1) y CV_G_ = 5,7%(4,7–10,6); para HbA_1c_, CV_I_ = 1,2%(0,3–2,5), CV_G_ = 5,4%(3,3–7,3) y para glucosa, CV_I_ = 5,0%(4,1–12,0), CV_G_ = 8,1%(2,7–10,8) no difirieron de los estimados globales previamente descritos.

**Conclusiones:**

La evaluación crítica y clasificación de los estudios de VB a tenor de su calidad metodológica, seguido de un meta-análisis, genera estimados de VB robustos y fiables. Este estudio proporciona datos de VB para albúmina glicolisada, glucosa y HbA_1c_ actualizados y basados en la evidencia científica.

## Introducción

La variación biológica (VB) se define como la fluctuación aleatoria de la concentración de una magnitud en un fluido biológico, debida a un equilibrio entre el recambio metabólico y la regulación homeostática [1], [2]. Tiene dos componentes, intra- e interindividual, que se expresan como coeficientes de variación (CV_I_ y CV_G_, respectivamente) [3]. Fraser y Harris publicaron un modelo para su estimación en 1989 [1], que ha sido utilizado de una forma más o menos rigurosa a lo largo de los años. En 1999 la Comisión de Calidad Analítica de la SEQC^ML^ [4] compendió todas las publicaciones que contenían estimados de la VB en una base de datos que se fue actualizando cada dos años hasta 2014 [5], siguiendo un criterio de inclusión de artículos que ha sido descrito en detalle por Perich y colaboradores [6].

A pesar de su innegable prestigio y difusión, en la última década diversos autores plantearon ciertas limitaciones de los estimados de VB contenidos en la base de datos de Ricós y cols [7], [8], [9]. Por ello, el *Working Group on Biological Variation* de la *European Federation of Clinical Chemistry and Laboratory Medicine* (EFLM WG-BV) desarrolló una herramienta de evaluación crítica denominada *Biological Variation Data Critical Appraisal Checklist* (BIVAC) [10]. Los estudios de VB incluidos en la base de datos hasta 2014, así como los encontrados en búsquedas bibliográficas posteriores, fueron revisados y calificados por el *Task Group of Biological Variation Database* (TG-BVD) utilizando la herramienta BIVAC; posteriormente se aplicó un método de meta-análisis para la obtención de nuevos estimados más robustos. Recientemente se han publicado los resultados obtenidos para magnitudes relacionadas con la diabetes mellitus (DM) [11], lípidos [12] y parámetros hematológicos [13].

Todo ello permitió crear una nueva base de datos de estimados de VB, cuyo lanzamiento tuvo lugar en el 23º congreso internacional de Laboratorio Médico *Euromedlab* 2019, que se encuentra disponible en https://biologicalvariation.eu/ [14]. Hasta hoy se han publicado estimados globales de VB, derivados de meta-análisis, para más de 100 mensurandos y continuamente se están añadiendo nuevos datos. Por ello, esta base de datos facilita información fiable para los mensurandos más frecuentemente analizados en los laboratorios clínicos, para la gestión y seguimiento de las enfermedades de alta prevalencia.

La Diabetes Mellitus (DM) es una de las patologías con mayor prevalencia en la población mundial (8,5%) [15] y existen guías y consensos internacionales sobre cómo llevar a cabo el diagnóstico y la monitorización del control glucémico. Uno de los marcadores que repunta en interés en los últimos años es la albúmina glicosilada [16], [17], [18], por su capacidad de monitorizar el metabolismo de la glucosa a largo plazo en situaciones que pueden interferir con el metabolismo de la hemoglobina. En la última década se han publicado numerosos artículos sobre VB de mensurandos relacionados con la diabetes y nuestro grupo realizó, previa evaluación crítica, una revisión sistemática que fue publicada en 2019 [11]. Sin embargo, no se incluyó entonces la albúmina glicosilada porque en aquel momento solo se disponía de un artículo publicado. Tampoco se había publicado el estudio multicéntrico europeo de alta fiabilidad (EuBIVAS) [18]. El objetivo de este estudio es doble. Por un lado, realizar una revisión sistemática y evaluación critica de estudios de VB para albúmina glicosilada y por otro, actualizar estimados de VB para glucosa y HbA_1c_, tras incluir estudios recientes como EuBIVAS [19].

## Materiales y métodos

Se realizó una búsqueda o revisión bibliográfica, sin limitación en el tiempo, de estudios sobre VB de albúmina glicosilada, y, de forma complementaria, otra búsqueda, acotada de Junio 2018 a Diciembre 2019, de nuevas publicaciones sobre VB de glucosa y HbA_1c_ no incluidas en la primera revisión [11], usando los mismos términos y palabras clave.

La herramienta de lectura crítica BIVAC [10] consta de 14 ítems, que clasifican los estudios de VB en 4 categorías A, B, C o D, en orden decreciente de calidad en relación al cumplimiento de los ítems

El ítem 1 verifica la escala del mensurando. Los ítems 2 a 4 verifican si los sujetos, muestras y métodos de medida, respectivamente, se describen con suficiente detalle. Los ítems 5 a 7 se refieren a las condiciones pre-analíticas y al estado de equilibrio homeostático de los sujetos estudiados. Los ítems 8 a 12 evalúan los métodos de medida y los cálculos estadísticos utilizados para obtener los estimados de CV_I_ y CV_G_. Para cumplir los ítems 13 y 14 se requiere la descripción del número de resultados incluidos en los cálculos realizados y de la concentración de la magnitud, respectivamente.

La clasificación global de los estudios de VB mediante BIVAC se establece en función de la puntuación mínima obtenida en cualquiera de los ítems. El cumplimiento total de los 14 ítems otorgará al estudio la clasificación A. Si no se cumplen los ítems esenciales (del 2 al 4), el estudio será clasificado como D.

El cálculo de los estimados globales de CV_I_ y CV_G_, para cada mensurando, se realizó mediante un meta-análisis [20] en el que se empleó la mediana ponderada de los valores de CV_I_ y CV_G_ obtenidos en cada estudio (A = 4, B = 2; C = 1), junto con la inversa de la amplitud del IC del CV_I_ y CV_G_ de cada estudio. Los intervalos de confianza al 95% (IC 95%) de los estimados globales se calcularon mediante un método de *boostrapping* con corrección de sesgo [21].

En el meta-análisis (realizado tanto con los artículos identificados en la primera revisión como con los encontrados en esta segunda revisión), se incluyeron los estudios que cumplían los siguientes criterios: grado BIVAC de A a C, realizados en adultos (18–75 años) sanos, con más de tres sujetos y más de 3 muestras por sujeto obtenidas en un intervalo comprendido entre dos extracciones semanales y una extracción mensual. Además, los estudios tenían que mostrar resultados de VB descritos en detalle e informar el coeficiente de variación analítico (CV_A_). Cuando una publicación proporcionaba datos de VB estratificados (por edad, sexo, etc.), y también globalizados, se tuvieron en cuenta solo estos últimos para evitar datos duplicados.

## Resultados

La búsqueda bibliográfica identificó 6 artículos; 2 de ellos publicados con posterioridad a BIVAC [10] (entre marzo 2018 y diciembre 2019) y 4 fueron estudios sobre albúmina glicosilada. En la Tabla 1 se muestran los artículos y mensurandos revisados, el año de publicación y la identificación numérica de los mismos.

**Tabla 1:** Artículos revisados en este estudio.

Año	Artículo	Magnitud	n
2019	Liang L, He H, Zeng Y, Zhang M, Wang X, Li X, Liang S et al. Evaluation of biological variation of glycated hemoglobin and glycated albumin in healthy Chinese subjects. J Clin Lab Anal 2019;33:e22715. https://doi.org/10.1002/jcla.2275.	Albúmina glicosilada HbA_1c_	501
2018	Aarsand AK, Diaz-Garzón J, Fernandez-Calle P, Guerra E, Locatelli M, Bartlett WA et al. The EuBIVAS: Within- and between-subject biological variation data for electrolytes, lipids, urea, uric acid, total protein, total bilirubin, direct bilirubin, and glucose. Clin Chem 2018;64:1380–1393.	Glucosa	335
2015	Parrinello CM, Lutsey PL, Couper D, Eckfeldt JH, Steffes MW, Caresh J et al. Total short-term variability in biomarkers of hyperglycemia in older adults. Clin Chem 2015;61:1540–1548	Albúmina glicosilada	278
2013	Montagnana M, Paleari R, Danese E, Slvagno GL, Lippi G, Giuidi GC et al. Evaluation of biological variation of glycated albumin (GA) and fructosamine in healthy subjects. Clin Chim Acta 2013;423:1–4	Albúmina glicosilada	273
2012	Xue L, Liang H, Jiang X. Circanual temperature-related variation in HbA_1c_ is unlikely to affect its use as a diagnostic test for type 2 Diabetes. Clin Lab 2012;58:481–488.	Glucosa	307
1993	Davie SJ, Whiting KL, Gould BJ. Biological variation in glycated proteins. Ann Clin Biochem 1993;30:260–264	Albúmina glicosilada	31

En la Tabla 2 se muestra el número de artículos revisados y su clasificación BIVAC. El mensurando con mayor número de publicaciones fue la glucosa. La mayor parte de los estudios se clasificaron como grado C.

**Tabla 2:** Número de artículos revisados, y clasificación BIVAC para artículos con estimados de VB para mensurandos relacionados con la DM.

Mensurando	n	Clasificación BIVAC			
		A	B	C	D
Albúmina glicosilada	4	0	1	3	0
HbA_1c_	1	0	1	0	0
Glucosa	2	1	0	1	0
Revisión sistemática previa [11]					
Albúmina glicosilada	NE	NE	NE	NE	NE
HbA_1c_	17	1	2	10	4
Glucosa	23	2	1	20	0

n, número de artículos revisados; NE, no estudiado.

La Tabla 3 contiene los estimados globales de CV_I_ y CV_G_ resultantes del meta-análisis realizado considerando conjuntamente los datos de las dos revisiones.

**Tabla 3:** Estimados globales de los componentes de VB.

Mensurando	CV_I_% (IC 95%)	CV_G_% (IC 95%)
Albúmina glicosilada	1,4 (1,2–2,1)	5,7 (4,7–10,6)
HbA_1c_	1,2 (0,3–2,5)	5,4 (3,3–7,3)
Glucosa	5,0 (4,1–12,0)	8,1 (2,7–10,8)

CV_I_, coeficiente de variación intra-individual; CV_G_, coeficiente de variación inter-individual; IC 95%, intervalo de confianza al 95%.

**Figura 1:**
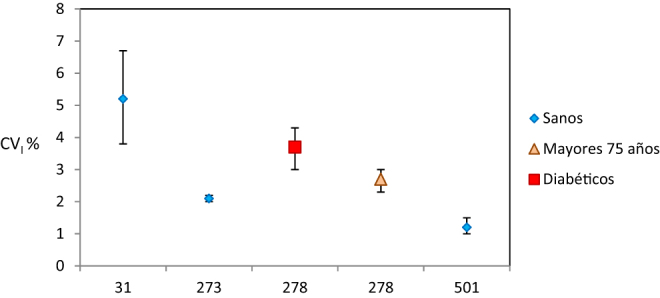
CV_I_ y sus intervalos de confianza para albúmina glicosilada de los artículos incluidos en esta revisión. Eje abcisas: número de identificación del artículo, acorde con la Tabla 1.

La Figura 1 muestra los CV_I_ de albúmina glicosilada, mensurando que no se había estudiado en la primera revisión [11]. En cuanto al CV_G_, solo tres artículos facilitan esta información, siendo el valor del artículo más reciente inferior a los de los más antiguos: CV_G_ = 4,7%, 10,3% y 10,7%, respectivamente.

## Discusión

Dada la relevancia clínica de las magnitudes relacionadas con la DM (debido a su papel clave en el diagnóstico y monitorización de la enfermedad), es esencial disponer de datos de VB robustos para las magnitudes utilizadas en el diagnóstico y monitorización de las patologías, porque de ellos derivan las especificaciones para imprecisión y sesgo, que ayudan a asegurar óptimas prestaciones analíticas, así como el valor de referencia del cambio, que permite realizar una adecuada interpretación de los resultados seriados [2], [22].

Para el diagnóstico y monitorización del control glucémico, clásicamente se utilizan la glucosa y la HbA_1c_ aunque actualmente algunos autores consideran la albúmina glicosilada como mejor magnitud que la HbA_1c_ para el diagnóstico, prevención de riesgo de DM y monitorización de pacientes diabéticos en situaciones clínicas que pueden interferir en el metabolismo de la hemoglobina [16], [17], [18]. De ahí la importancia de tener estimados robustos de BV disponibles para los laboratorios que adopten esta estrategia de monitorización.

La revisión actual muestra que los estudios encontrados presentan prácticamente las mismas debilidades respecto al modelo descrito en BIVAC, y ya evidenciado en la revisión anterior [11]. En resumen, de los 75 estudios (primera y segunda revisión), la mayor parte se clasifican como C, independientemente del mensurando: el 86% no realiza el análisis de valores extremos o aberrantes (ítem 8), el 64% carece de información relativa al estudio de la homogeneidad de las varianzas (ítem 10), el 50% no indica el intervalo de confianza de los estimados de VB o no hace posible su cálculo, ni cuantifica el número de resultados excluidos (ítems 12 y 13, respectivamente), el 60% en la primera revisión y el 30% en la segunda calcula el CV_A_ a partir de los resultados del control interno de la calidad, en lugar de utilizar los replicados de las muestras obtenidas de los participantes (ítem 6), y el 20% no comprueba el equilibrio homeostático de los sujetos durante el período de tiempo estudiado (ítem 7).

Estas características merman valor a los estimados obtenidos por los diferentes autores en sus estudios. Así, la ausencia de análisis de valores aberrantes (ítem 8 de BIVAC) y de la homogeneidad de varianzas (ítem 10) puede tener como consecuencias la obtención de estimados de CV_I_ y CV_G_ no transferibles o poco fiables.

La no indicación del número de resultados finales incluidos en el cálculo del estimado (ítem 13), dificulta la evaluación de la homogeneidad de la cohorte seleccionada y de la transferibilidad y la robustez de los datos generados.

BIVAC establece como requisito de calidad que todos los estudios de VB incluyan la estimación del CV_A_ a partir de replicados de las muestras de los sujetos estudiados (ítem 6) [1], [10].

Otro aspecto muy importante es comprobar el equilibrio homeostático de los mensurandos en los sujetos estudiados (ítem 7), debido a que variaciones en la concentración podrían influir en la estimación de los valores de CV_I_ y CV_G_.

Una de las principales fortalezas del BIVAC es que el meta-análisis empleado para obtener los estimados globales de CV_I_ y CV_G_ prioriza y da más peso a los estudios de calidad óptima (clasificación A).

En esta segunda revisión se identificaron dos nuevos artículos para glucosa, que cumplen los requisitos de inclusión en el meta-análisis para actualizar los estimados globales; éstos no difieren de los publicados en la primera revisión (Tabla 3).

Para la HbA_1c_: los estimados de CV_I_ y CV_G_ son muy similares a los publicados previamente [11], con un IC más estrecho probablemente debido a la inclusión de un nuevo artículo de grado B [23] (número 501 en la Tabla 1).

La revisión de los datos de albúmina glicosilada evidencia una considerable dispersión entre los cuatro CV_I_ de los estudios realizados en adultos sanos; los dos estudios recientes (números 273 [24] y 501 [23], de 2013 y 2019, respectivamente) mostrando CV_I_ más bajos (Figura 1). Un estudio realizado en sujetos mayores de 75 años y en un grupo de pacientes diabéticos (número 278 en la Figura 1) [25] mostró estimados significativamente superiores.

El alto CV_I_ obtenido en el artículo más antiguo (de 1993) (número 31 en la Figura 1) [26] es debido probablemente al empleo de un método analítico en desuso (cromatografía de afinidad), comparado métodos más específicos (enzimático automatizado) utilizados en los otros estudios.

El estudio (número 501) [23] está realizado en población china y muestra un CV_I_ menor, debido quizás a la distinta etnicidad.

Estos estimados CV_I_ = 1,4% (1,2–2,1), CV_G_ = 5,7% (4,7–10,6) difieren remarcablemente de los contenidos en la base de datos del año 2014 [5] (CV_I_ = 5,2% and CV_G_ = 10%), donde solo se incluían datos del primer estudio basado en cromatografía de afinidad. Harían falta más estudios de alta calidad para producir estimados fiables de CV_I_ y CV_G_ para albúmina glicosilada.

BIVAC se publicó en marzo de 2018. En esta revisión solo se han encontrado dos estudios publicados con posterioridad. Estos dos artículos, uno para glucosa y otro para albúmina glicosilada y HbA_1c_, obtuvieron el grado A (número 335 en la Tabla 1) [19] y B (número 501 en la Tabla 1) [23], respectivamente, lo que parece indicar que los autores basan el diseño y análisis de datos de sus estudios en BIVAC. Sin embargo, uno de estos estudios es EuBIVAS, publicado por el WG-BV, que usó este diseño incluso antes la publicación de BIVAC. En consecuencia, como uno de los objetivos de BIVAC es mejorar la calidad de los estudios futuros, es todavía demasiado pronto para concluir si este objetivo se ha cumplido.

## Conclusiones

La aplicación de la herramienta BIVAC para la evaluación de estudios de VB es un método objetivo y estandarizado que permite obtener estimados de VB robustos y fiables. En nuestro estudio hemos producido estimados globales de VB para albúmina glicosilada basados en la evaluación crítica y meta-análisis de estudios relevantes. La inclusión de trabajos recientemente publicados para glucosa y HbA_1c_ no cambia el estimado global presentado en nuestra revisión previa. Sin embargo, la disponibilidad de mayor proporción de estudios de alta calidad en el futuro puede tener un impacto mayor y es probable que el IC decrezca progresivamente.

El interés de BIVAC no sólo se limita a la evaluación de estudios ya publicados, sino que su empleo permitirá a futuros investigadores disponer de un estándar internacional para el diseño, realización y publicación de estudios sobre VB, así como de guía de referencia en el diseño de nuevos estudios de VB.

El escaso número de estudios que obtienen la mejor clasificación BIVAC demuestra la necesidad de disponer de más trabajos, que cumplan los requisitos de la herramienta BIVAC para conseguir estimados robustos de VB. La aplicación de la herramienta BIVAC en el diseño y ejecución de futuros estudios probablemente mejorará la calidad/fiabilidad de los estimados de VB.
